# Bronchus-Associated Lymphoid Tissue (BALT) and Survival in a Vaccine Mouse Model of Tularemia

**DOI:** 10.1371/journal.pone.0011156

**Published:** 2010-06-16

**Authors:** Damiana Chiavolini, Javier Rangel-Moreno, Gretchen Berg, Kate Christian, Laura Oliveira-Nascimento, Susan Weir, Joseph Alroy, Troy D. Randall, Lee M. Wetzler

**Affiliations:** 1 Department of Medicine, Boston University School of Medicine, Boston, Massachusetts, United States of America; 2 Division of Allergy, Immunology and Rheumatology, University of Rochester Medical School, Rochester, New York, United States of America; 3 School of Pharmaceutical Sciences, University of São Paulo, São Paulo, Brazil; 4 Department of Pathology, Tufts University School of Medicine, Cummings School of Veterinary Medicine and Tufts Medical Center, Boston, Massachusetts, United States of America; Louisiana State University, United States of America

## Abstract

**Background:**

*Francisella tularensis* causes severe pulmonary disease, and nasal vaccination could be the ideal measure to effectively prevent it. Nevertheless, the efficacy of this type of vaccine is influenced by the lack of an effective mucosal adjuvant.

**Methodology/Principal Findings:**

Mice were immunized via the nasal route with lipopolysaccharide isolated from *F. tularensis* and neisserial recombinant PorB as an adjuvant candidate. Then, mice were challenged via the same route with the *F. tularensis* attenuated live vaccine strain (LVS). Mouse survival and analysis of a number of immune parameters were conducted following intranasal challenge. Vaccination induced a systemic antibody response and 70% of mice were protected from challenge as showed by their improved survival and weight regain. Lungs from mice recovering from infection presented prominent lymphoid aggregates in peribronchial and perivascular areas, consistent with the location of bronchus-associated lymphoid tissue (BALT). BALT areas contained proliferating B and T cells, germinal centers, T cell infiltrates, dendritic cells (DCs). We also observed local production of antibody generating cells and homeostatic chemokines in BALT areas.

**Conclusions:**

These data indicate that PorB might be an optimal adjuvant candidate for improving the protective effect of *F. tularensis* antigens. The presence of BALT induced after intranasal challenge in vaccinated mice might play a role in regulation of local immunity and long-term protection, but more work is needed to elucidate mechanisms that lead to its formation.

## Introduction

*Francisella tularensis* is a gram-negative bacterium, and the cause of a severe pneumonic disease known as tularemia. Although the number of cases of respiratory tularemia is relatively low worldwide, the potential for using this organism as a biological weapon has encouraged the search for an effective vaccine. Nasal immunization is a promising alternative to classical parenteral vaccination, because it is non-invasive and capable of eliciting both systemic and local immune responses. In addition, this vaccination route is known to be more immunogenic at the mucosal level than the oral and vaginal routes [1], [2]. Another advantage is that it requires smaller amounts of antigen to induce an optimal immune response [3], [4]. Nevertheless, the development of mucosal vaccines is generally limited by the lack of effective mucosal adjuvants [5], [6].

With regard to intranasal vaccines against tularemia, live organisms have mostly been tested via this route, conferring variable levels of protection against challenge with virulent *Francisella*. Examples of these immunogens are LVS, attenuated *Francisella novicida* strains and mutants of the virulent SchuS4 strain [7]–[10]. Although the live vaccine strain (LVS) of *F. tularensis* derived from a virulent type B strain has been used for vaccination, it is no longer approved for human use because the basis for its attenuation still remain obscure [11]. Attractive and safe alternatives to substitute live organisms are subunit vaccines, though their use against tularemia has not been fully investigated. Even more attractive could be the use of subunit vaccines for nasal immunization, to induce mucosal protection against tularemia in a safer and potentially more effective way, although this approach has not been widely explored. Our group has previously shown that lipopolysaccharide (LPS) from *F. tularensis* in combination with porin B (PorB) purified from *Neisseria meningitidis* elicited 70% protection from bacterial challenge, when given subcutaneously [12]. Other groups have reported that mice immunized with *F. tularensis* LPS via several systemic routes were marginally protected against intraperitoneal and intradermal challenge with type B strains [13]–[17]. Several proteins, including a 17-kDa protein (Tul4), a 43-kDa outer membrane protein and heat shock protein 60 have been tested for their efficacy in animal models, but they conferred minimal protection after challenge with virulent strains [18]–[20]. One group reported that immunization with native outer membrane proteins (OMPs) induced 50% protection against intranasal challenge with type A *F. tularensis* [21]. More recently, a detoxified endotoxin (from *Escherichia coli*) vaccine non covalently complexed with the outer membrane protein of *N. meningitidis* group B was also found to be partially protective against LVS and type A virulent strains [22].

Overall, research efforts to investigate novel mucosal adjuvants that potentiate the response to *F. tularensis* antigens have been minimal. One study reported the use of cholera toxin subunit B (CTB) as a nasal adjuvant with inactivated LVS against both LVS and virulent *F. tularensis* [23].

Bronchus-Associated Lymphoid Tissue (BALT) is a lymphoid structure that can be found in peribronchial, perivascular and interstitial areas of the lung. Its formation can be triggered in the lungs of mice and humans by encounter with antigen, infection or inflammation, but it is not normally present in healthy lungs of these species [24]. BALT is composed of prominent lymphocyte aggregates, often characterized by proliferating and germinal center B cells, supported by a central follicular dendritic cell network. Interfollicular T cells and dendritic cells lie underneath the follicle associated epithelium (FAE) and are located around B cell areas [25], [26]. Other important constituents of this specialized lymphoid tissue are lymphatics and high endothelial venules (HEVs) expressing vascular cellular-adhesion molecule-1 (VCAM-1) [27], [28].

It has been reported that similar structures were formed as a direct consequence of certain respiratory infectious diseases in experimental animal models. The influenza virus triggered formation of what is known as inducible BALT (iBALT) in mice lacking conventional lymphoid organs. It was suggested that iBALT may play an important role in protection [27], [29]. Also, lungs of several other animal species infected either naturally or experimentally with a number of bacterial and viral pathogens also developed areas of organized lymphoid follicles [30]–. Lungs of patients with pulmonary complications of Sjogren's syndrome (SS) and rheumatoid arthritis (RA) showed areas of organized lymphoid areas, also referred to as iBALT [34]. Despite having a critical role in the modulation of local inflammatory response in mice inoculated with Influenza (JRM personal communication), the specific function of iBALT in infection and immunity still remains controversial, considering that this tissue only develops as a consequence of certain infectious diseases but not others.

The present study reports the potential use meningococcal recombinant porin B (rPorB) as a putative mucosal adjuvant candidate, when delivered with *F. tularensis* LPS. The formation of highly organized BALT, following intranasal vaccination and subsequent bacterial challenge is also described.

## Results

### Induction of systemic antigen specific antibodies after vaccination

**Figure 1:**
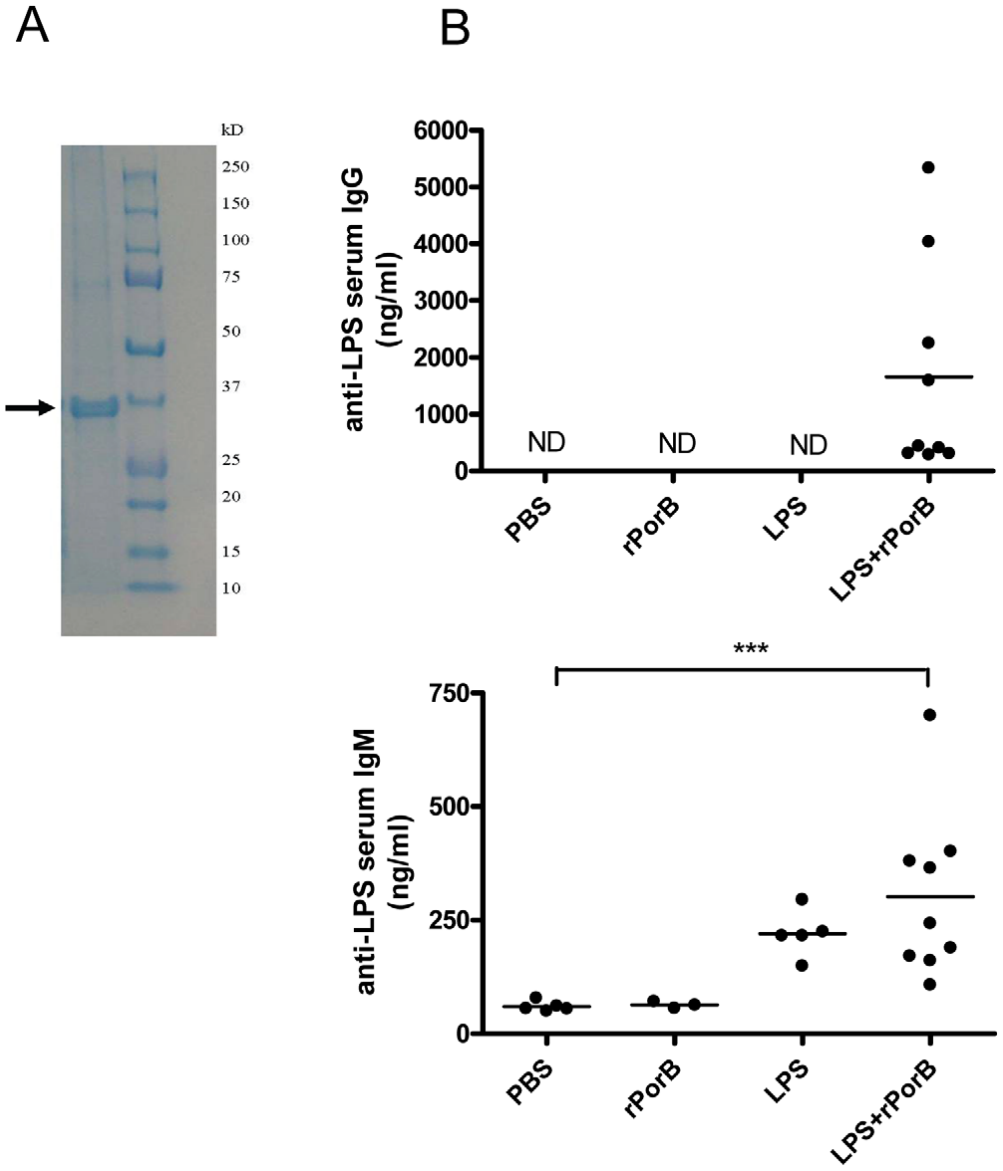
Recombinant PorB and evaluation of antibody response against *F.tularensis* LPS. (A) The band of rPorB detected by Coomassie gel stain is indicated by the arrow. (B) Serum was collected four weeks after vaccination, just before i.n. challenge. Antibody production was analyzed by ELISA and results are presented as nanograms (ng) of LPS-specific IgG and IgM per milliliter (ml) of serum. Black dots indicate individual mice, while bars represent mean values. Lower antibody concentrations include values between 286 and 436 ng/ml as opposed to those that were not detected (ND) in control groups. Significant differences were calculated by Mann Whitney test and are indicated by *** (*P*<0.001) for LPS+rPorB compared with PBS.

SDS-PAGE was used to detect purified rPorB, as shown by the single band in the Coomassie gel (Figure 1A). Minimal endotoxin levels, approximately 0.036 EU per microgram, were detected in the protein preparation. After confirming the purity of rPorB, the humoral response to intranasal vaccination with the LPS+rPorB candidate was assessed. Blood was collected from all groups four weeks after the third immunization dose and just before intranasal challenge. Serum levels of antigen specific antibodies were measured by ELISA. Overall, mice that had received LPS+rPorB via the nasal route developed higher levels of LPS-specific immunoglobulins than control groups (Figure 1B). Sera from mice vaccinated with LPS+rPorB contained antigen specific IgG, though levels of this immunoglobulin were shown to be variable, with four mice responding more strongly than the other five (Figure 1B). The variability in IgG response did not necessarily reflect the outcome of survival following intranasal challenge. No detectable levels of IgG were present in serum from mice in the PBS, rPorB and LPS alone control groups. Levels of LPS-specific IgM detected in the serum of mice vaccinated with LPS/rPorB were overall higher than those observed in mice immunized with control substances, with a significant difference when compared with the PBS group (Figure 1B). As for IgG, mouse to mouse variability in the IgM response was also observed. Analysis of serum antigen-specific IgA, revealed that only two out of 10 mice vaccinated with LPS+rPorB had increased antibody levels when compared to the three control groups (data not shown).

### Nasal delivery of rPorB improved the protective capacity of LPS against *F. tularensis* challenge

**Figure 2:**
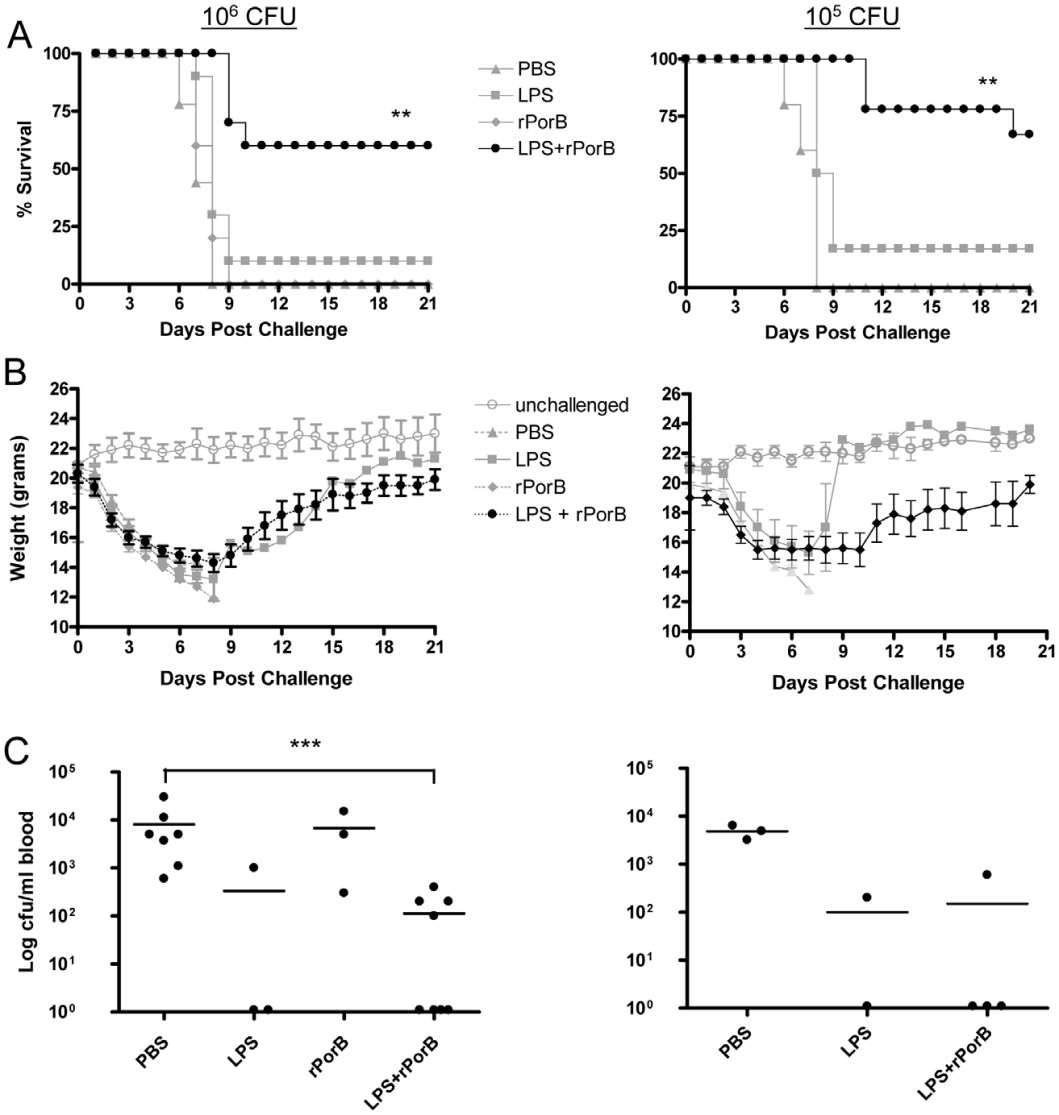
Survival, morbidity and bacterial dissemination following intranasal challenge. (A) Protection was monitored for 21 days in challenged groups after inoculation with 10^6^ and 10^5^ CFU of LVS. (B) Morbidity associated with bacterial infection was determined by analyzing changes in body weight. The weight regain curve for the LPS-vaccinated group includes the only mouse that survived intranasal challenge (filled grey squares). (C) Bacterial loads in blood of challenged mice. Black dots represent individual mice, while bars represent mean values. ** (*P*<0.01); *** (*P*<0.001).

To determine the protective capacity of *F. tularensis* LPS with rPorB administered intranasally, we challenged groups of mice with either 10^5^ CFU (100× LD_50_) or 10^6^ CFU (1000× LD_50_) of LVS via the same route. Vaccinated and control animals received the bacterial inoculum four weeks after the third immunization and their survival was monitored for 21 days. Sixty percent of the mice (6 out of 10) immunized with the LPS+rPorB were protected from challenge with 10^6^ CFU of LVS, compared with the PBS control group that succumbed to tularemia within 8 days post-infection (Figure 2A). The poor protective capacity of LPS alone was reflected in the accelerated death of mice (Figure 2A). After infection with 10^5^ CFU, 7 mice out of 9 (78%) of the LPS+rPorB group survived for 19 days (Figure 2A). At day 20 one additional mouse from the 10^5^ CFU group died with a final survival rate of 67% (Figure 2A). In contrast, only 10% of inoculated with LPS alone survived after challenge with 10^6^ CFU, and 17% survived after challenge with 10^5^ CFU (Figure 2A). Progressive body weight loss was detected during the first 8 days after infection, and a subsequent regain, starting at day 9 post-infection, was associated with improved survival of LPS+rPorB vaccinated mice (Figure 2B). Mice that received LPS alone and survived challenge had a similar body weight recovery, although the overall survival rate was dramatically reduced compared to LPS+rPorB mice. Groups of mice receiving PBS or rPorB showed poor survival rate and never regained their initial weight. To further evaluate the efficacy of intranasal vaccination, bacterial replication in the bloodstream was analyzed. Mice (3 to 8 mice per group) immunized with LPS+rPorB or one of the control substances (PBS, LPS or rPorB alone) were bled 3 days after intranasal challenge with either 10^5^ or 10^6^ CFU of LVS. Figure 2C shows that mice immunized with our vaccine candidate (LPS+rPorB) had numbers of bacteria in their blood (∼10^2^ CFU per ml) approximately 100-fold lower than mock-vaccinated mice (∼10^4^ CFU; *P*<0.001). Bacteremia detected in LPS-immunized mice was similar to that observed in the LPS+rPorB-immunized group, while bacterial levels in the blood of mice vaccinated with rPorB were closer to those found in PBS control mice (Figure 2C). A similar pattern was observed in groups of mice challenged intranasally with 10^5^ CFU (Figure 2C). These results describe the high potential of meningococcal porin as a mucosal adjuvant. In a preliminary study we compared the immunogenic efficacy of this protein to that of the known mucosal adjuvant CpG DNA. Results revealed that LPS-specific IgG and IgM induced by PorB were respectively 3 and 2-fold higher when compared with CpG DNA (data now shown).

### Formation of BALT after vaccination and challenge

**Figure 3:**
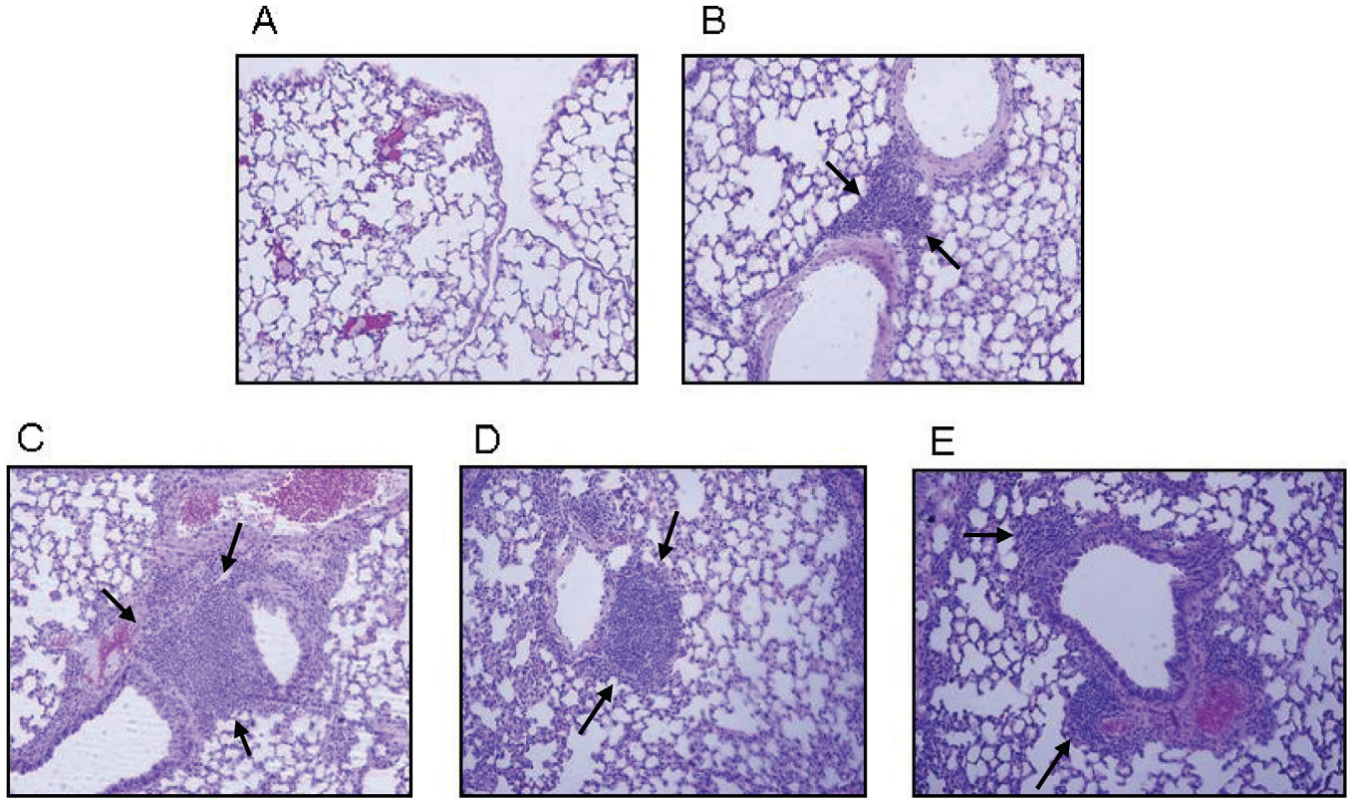
Lymphoid aggregates in lungs of vaccinated mice were detected at 30 days post-challenge (arrows). (A) Representative lung from naïve mouse was used as a negative control; (B) Lung from LPS vaccinated/challenged; (C and D) Lung from LPS+rPorB vaccinated/challenged mouse and (E) Lung from LPS+rPorB vaccinated/unchallenged mouse. Formalin fixed, paraffin embedded sections were stained with H&E and representative pictures were taken with a 10× objective.

**Figure 4:**
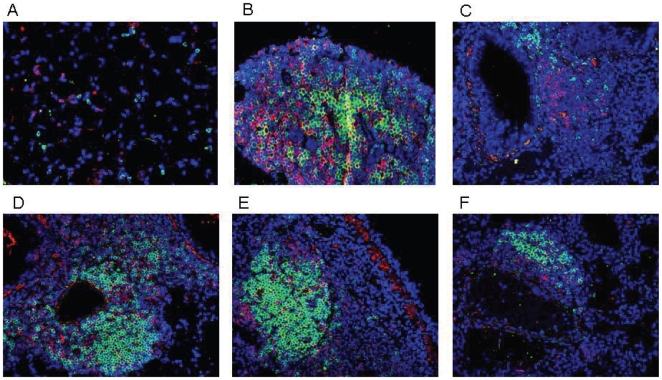
Detection of B and T cell infiltrates in lungs of vaccinated mice 30 days post-challenge. (A) Representative lung from naïve mouse as a negative control; (B) Mediastinal lymph node from LPS+rPorB/challenged mouse as an internal positive control; (C) Lung from LPS vaccinated/challenged mouse; (D, E) Lung from LPS/rPorB vaccinated/challenged mouse and (F) lung from LPS+PorB vaccinated/unchallenged mouse. Cell surface markers used were B220 for B cells (green- Alexa 488) and CD3 epsilon for visualizing T cells (red- Alexa 549). Cell nuclei were stained with 4′-6-Diamino-2-phenylindole (DAPI-blue). Representative pictures were taken with a 20× objective.

**Figure 5 pone-0011156-g005:**
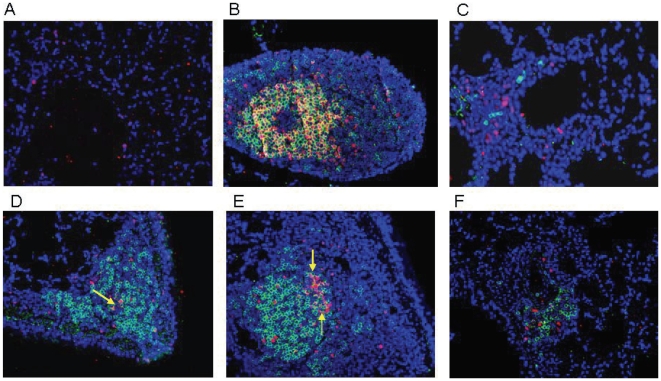
Detection of proliferating B cells within BALT of vaccinated mice 30 days post-challenge. (A) Lung from naïve mouse as a negative control; (B) mediastinal lymph node from LPS+rPorB/challenged mouse as an internal positive control; (C) Lung from LPS vaccinated/challenged mouse; (D, E) Lung from LPS+rPorB vaccinated/challenged mouse and (F) lung from LPS+PorB vaccinated/unchallenged mice. B cells were detected with B220 (green- Alexa 488) and PCNA was used to identify proliferating cells (red- Alexa 549). Areas of mixed color overlap indicate proliferation of B cells (see arrows). Cell nuclei were stained with 4′-6-Diamino-2-phenylindole (DAPI-blue). Representative pictures were taken with a 20× objective.

**Figure 6:**
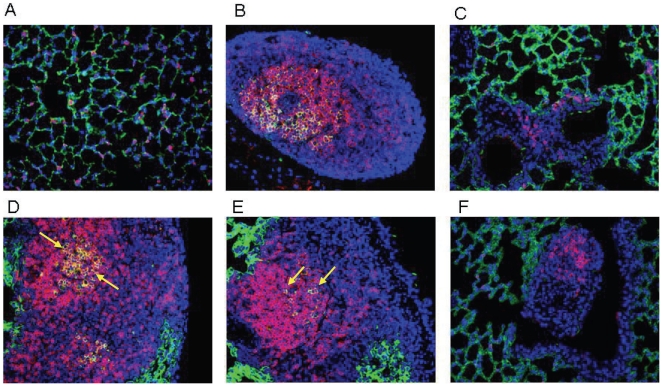
Germinal center B cells are exclusively found within BALT from LPS+rPorB vaccinated/LVS challenged mice. (A) Representative lung from naïve negative control; (B) mediastinal lymph node from LPS+rPorB/challenged mouse as an internal positive control; (C) Lung from LPS vaccinated/challenged; (D, E) Lung from LPS+rPorB vaccinated/challenged mouse and (F) lung from LPS+PorB vaccinated/unchallenged mouse. Cells markers are B220 for B cells (red- Alexa 549) and PNA to detect germinal center B cells (green- Alexa 488). Areas of color overlap indicate germinal centers (see arrows). Cell nuclei were stained with 4′-6-Diamino-2-phenylindole (DAPI-blue). Representative pictures were taken with a 20× objective.

Thirty days after respiratory challenge, lungs from vaccinated mice were dissected, fixed and processed for histopathological and immunofluorescence analyses. To evaluate the inflammatory changes in the infected lungs, paraffin sections were stained with hematoxylin and eosin (H&E). The presence of lymphocyte clusters, indicative of organized lymphoid follicles, was then evaluated by light microscopy. Representative lung sections from untreated mice (Figure 3A) as well as sections from lungs of mice vaccinated intranasally with LPS alone (Figure 3B), LPS+rPorB (Figure 3, C and D) and LPS+rPorB without LVS challenge (Figure 3E) are reported. Histopathological analysis of sections revealed the presence of lymphoid follicles. Overall, no inflammation (neutrophils, edema, fibrin) was detected in the lung tissue. Lungs from unvaccinated and unchallenged control mice did not contain any inflammatory cells or visible lymphocytes (Figure 3A). Lungs from vaccinated mice that became moribund prior to day 8 post-infection did not contain any lymphoid areas (data not shown). Lung sections from all vaccinated mice presented lymphoid aggregates (Figure B–E). Nevertheless, standard hematoxylin and eosin staining did not show the extent of the organization of the follicles, thus immunofluorescence was used for this purpose. Lung sections from the group vaccinated with LPS/rPorB and challenged with LVS contained visibly organized BALT as demonstrated by B (B220^+^) and T cell (CD3^+^) infiltration (Figure 4, D and E), B220^+^ B cell follicles with proliferating B cell (PCNA^+^) (Figure 5, D and E) and B220^+^ B cell clusters with peanut agglutinin-positive (PNA^+^) germinal center B cells (Figure 6, D and E). The lymphoid clusters detected in lungs from LPS+LVS challenged group and the LPS+rPorB unchallenged group were confirmed to be composed of disorganized B cells and some scattered T cells (Figures 4–6, C and F). Mediastinal lymph nodes were included in the study as positive staining controls (Figures 4–6, B). BALT was also detected in LPS+rPorB vaccinated at 120 days post-infection (data not shown). No viable organisms were detected in the lung, spleen and liver tissue with evident BALT at days 30 and 120 post-infection (data not shown), suggesting the formation of these structures may prevent dissemination of the infection.

### Morphometric analysis of lymphoid follicles and identification of dendritic cells, antibody producing cells and homeostatic chemokines in lungs

**Figure 7:**
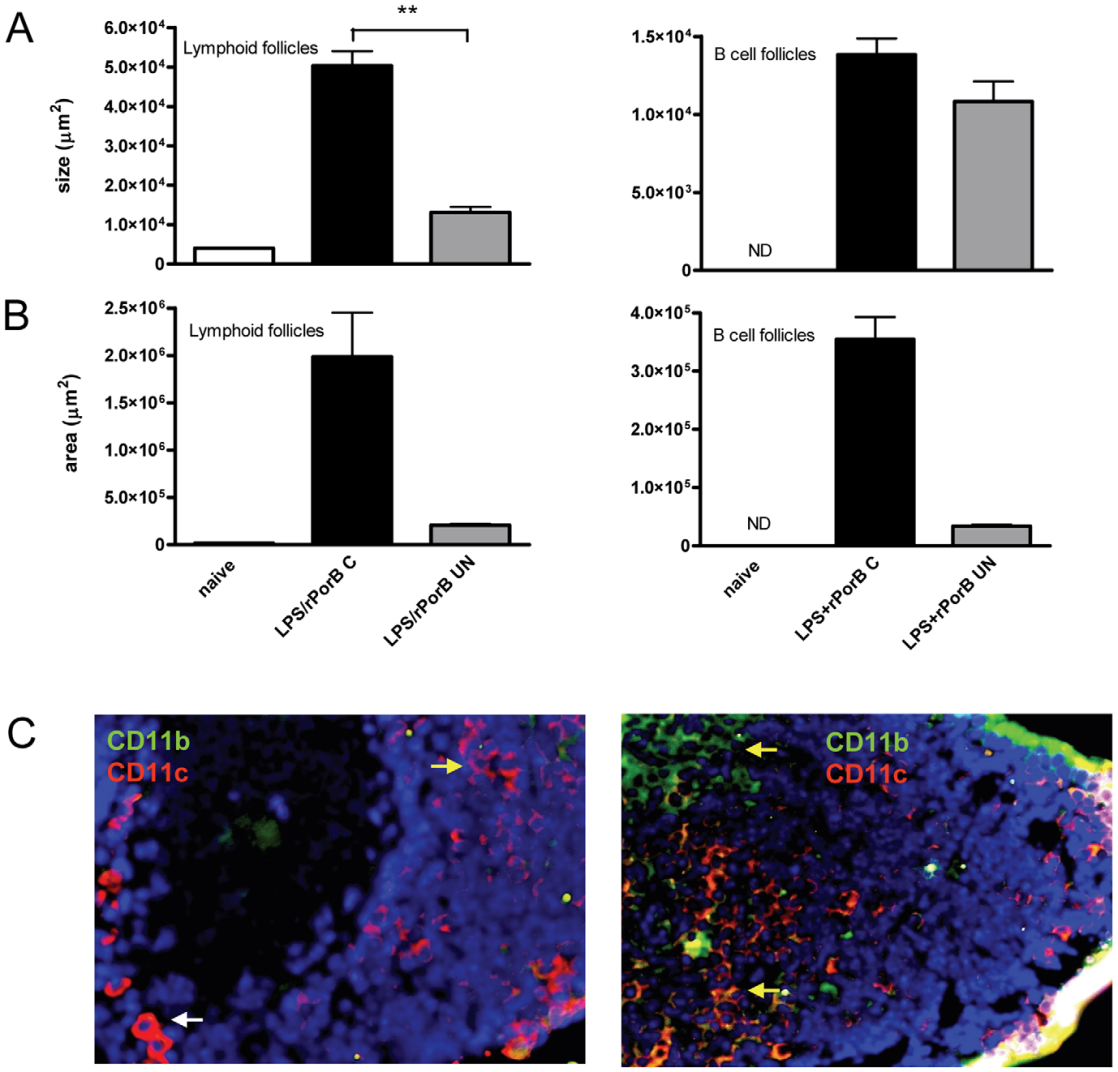
Morphometric analysis of follicles and pulmonary dendritic cells at 30 days post-challenge. Data are expressed as average size of (A) and area occupied by (B) lymphoid and B cell follicles (µm^2^). (C) Dendritic cells (DCs) are shown from one representative mouse immunized with LPS+PorB and challenged with LVS (the two panels show different fields of the same lung where cells were detected). DCs were detected within follicles by CD11b (green), CD11c (red) or both (color overlap) and characterized by cytoplasmic prolongations. DCs are indicated by the yellow arrows. The white arrow on the left panel indicates a macrophage, characterized by a rounded shape. ** (*P*<0.01). Sections were viewed with a 20× objective.

**Figure 8:**
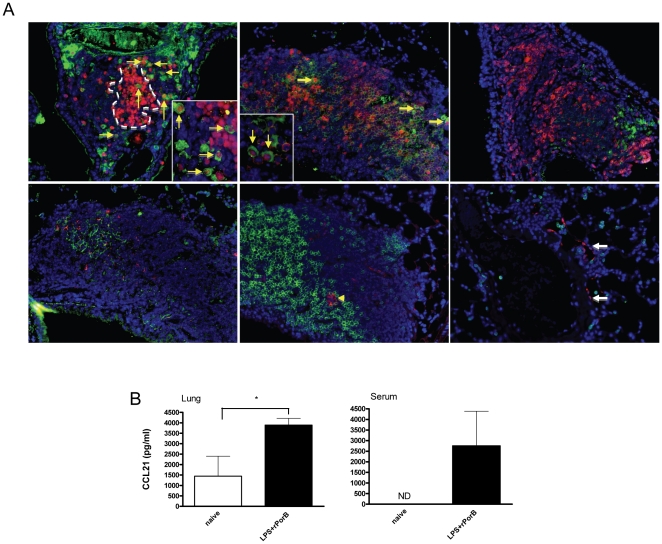
Antibody producing cells and homeostatic chemokines in BALT areas at 30 days post-challenge. (A) Representative lungs from mouse vaccinated with LPS+rPorB and challenged with LVS. The PCNA-CD3 combined stain was conducted to differentiate between nuclear (PCNA+) and surface (CD3+) staining. The upper left panel shows GC (cluster is outlined by the white dashed line) shown by the presence of cells with large PCNA+ nuclei (red nucleus staining). IgG producing proliferating plasma cells (indicated by the yellow arrows-main panel and insert) are detected with an anti-mouse IgG antibody (green cytoplasm) and PCNA (red nuclei), and few CD3+ cells (red surface) were identified in the same location. The upper middle panel shows IgM producing proliferating plasma cells (indicated by the yellow arrows-main panel and insert), identified by PCNA+ stain (red nuclei) and a dense IgM+ antibody signal (green cytoplasm). IgM+ B cells had a distinctive signal localized exclusively on the surface (green surface), and some CD3+ cells (red surface) were also detected. In the upper right panel, few IgA producing plasma cells (green cytoplasm) are present, but no proliferation is evident. Several CD3+ cells (red surface) were identified in the same area. In the lower left panel, some CXCL13 signal (red surface) co-localizes with the FDC network (combination of CD21, CD35 and FDCM1 markers-green) in the BALT area. The lower middle panel shows B cell clusters (green surface) and a CCL21+ high endothelial venule like structure (HEV) (red surface) as indicated by the yellow arrowhead. In the lower right panel, CCL21+ cells (red surface), resembling lymphatics or endothelial cells are shown by the white arrows. All sections were viewed with a 20× objective, and a 40× objective for the inserts in the first two upper panels. (B) Levels of CCL21 in lung lysate and serum are expressed as picograms (pg) per milliliter (ml) of lung lysate or serum. * (*P*<0.05).

To highlight differences in the inflammatory responses induced in the lung tissue of vaccinated/challenged mice and vaccinated/unchallenged mice 30 days after infection, morphometric analysis of lymphoid structures was conducted by calculating the average size of individual follicles, or the entire area occupied by multiple follicles. The average size of whole lymphoid follicles was significantly larger in lungs of mice vaccinated with LPS+rPorB and subsequently challenged (∼50,000 µm^2^), than in mice that were only vaccinated (∼13,000 µm^2^); differences were significant with a *P* value<0.01 (Figure 7A). B cell follicles were also slightly bigger in the vaccinated/challenged group (∼13,800 µm^2^) compared with mice that only received the vaccine (∼10,800 µm^2^), though this difference was not significant (Figure 7A). The total area occupied by lymphoid follicles was larger in the LPS+rPorB/challenged group (∼2,000,000 µm^2^) as compared to total area in LPS+rPorB/unchallenged group (∼207,000 µm^2^), although these differences were not statistically significant (Figure 7B). A similar pattern was observed for the average area occupied by B cell lymphoid aggregates that was increased in vaccinated/challenged mice (∼354,000 µm^2^) than in mice that were vaccinated but not challenged (∼33,800 µm^2^) (Figure 7B). Morphometric analysis for PBS- and rPorB-vaccinated groups was not conducted because all challenged mice had died by day 30, and no lymphoid aggregates were found in their lungs at the moribund stage. Results for the LPS-vaccinated group are also not shown because only one mouse per group survived bacterial challenge, and thus could not be included in the statistical analysis of the morphometric study. At day 30, dendritic cells (DCs) were found within the BALT follicles from mice vaccinated with LPS+PorB and challenged, as shown in figure 7C. Cells stained with either CD11b, CD11c, or a combination of both cell markers, were found and confirmed to be DCs by their typical appearance characterized by cytoplasmic prolongations (Figure 7C, yellow arrows). We were also able to identify CD11c+ high macrophages, featuring a rounder and more defined shape (Figure 7C, white arrow). We analyzed antibody generating plasma cells in lungs of challenged mice that had previously been vaccinated and euthanized at day 30. IgG+, IgA+ and IgM+ cells were detected in areas of BALT, and outside the lymphoid structures (data not shown). IgG+ plasma cells were more abundant than IgM+ and IgA+ plasma cells and some were proliferating (red nucleus stained by PCNA and green cytoplasm stained for IgG, indicated by the arrows in the upper left panel of figure 8A). IgM+B cells were also present, as shown by the signal localized exclusively on the surface (upper middle panel, figure 8A). No proliferating T cells were detected, but clusters of PCNA+ cells (red nuclei) indicating germinal centers (GC) and individual T cells (red surface) were found in the tissue (upper panels, figure 8A). The production of homeostatic chemokines, concomitant to the development of BALT in lungs of vaccinated mice, was analyzed locally by immunofluorescence and measured in serum and supernatants from lung homogenates, 30 days post-challenge. We were able to detect a CXCL13 signal that co-localized with follicular dendritic cells (detected by CD21, CD35 and FDCM-1 markers- lower left panel, figure 8A), and CCL21 signal in a structure presenting the characteristic morphology of a high endothelial venule, located in the middle of the B cell follicle (lower middle panel, figure 8A). CCL21 was also detected in cells with an oval morphology compatible with endothelial cells or lymphatic precursors (lower right panel, figure 8A). By ELISA assay, the levels of CCL21 in lung lysates and serum were elevated above baseline in the group vaccinated with LPS+rPorB and then challenged with LVS, as compared with unvaccinated unchallenged mice. A significant increase in CCL21 concentration in lung lysates from vaccinated mice was found (Figure 8B). Analysis of CXCL13 production in lung lysates and serum did not reveal any noteworthy differences between naïve and vaccinated mice (data not shown). Chemokine analysis is not reported for the LPS-vaccinated mice because only one mouse survived in the group, and data analyzed at day 30 could not be included in the study.

## Discussion

The present work reports the use of meningococcal PorB as a potential mucosal adjuvant when delivered with *F. tularensis* LPS, a poorly immunogenic antigen. A high percentage of mice vaccinated with LPS and PorB were significantly protected against lethal respiratory LVS infection when compared with the control groups, and presented well-organized bronchus-associated lymphoid tissue (BALT) in peribronchial and perivascular areas of their lungs. The vaccine candidate was administered via the intranasal route, an attractive alternative to conventional parenteral formulations against respiratory pathogens. We are aware that LVS is an attenuated strain of *F. tularensis*, and that challenge should ideally also be conducted with virulent type A and B strains. Nevertheless, it should be noted that very few studies have been able to show effective protection against virulent *F. tularensis* strains after administration of LPS-based or other subunit vaccines. Poor protection is associated with the rapid induction of fatal disease in mice challenged with extremely low infection doses. In addition to this experimental difficulty, virulent strains of *F.tularensis* cause fulminant systemic disease in the mouse, which does not optimally mimic human tularemia. Overall, the selection of a vaccine candidate able to confer successful protection against virulent *F. tularensis* has proven to be difficult over the years, and is beyond the purpose of the present work, which mostly shows the potential of a bacterial porin protein as a mucosal candidate and its ability to enhance BALT development.

Following intranasal immunization of mice, meningococcal recombinant PorB significantly improved the protective capacity of Ft-LPS, as shown by the increased survival rate of C57BL/6 mice (67% and 60%) challenged intranasally with 10^5^ (100× LD_50_) and 10^6^ CFU (1000× LD_50_) of LVS, respectively. LPS administered alone conferred less than 20% protection from respiratory challenge, confirming the results we previously obtained in a subcutaneous (s.c.) vaccination model [12]. In the s.c. model, LPS+PorB induced slightly higher protection (70%) than that observed in our i.n. model, but it should be noted that BALB/c mice were used instead of the current C57BL/6 strain [12], and that genetic background can have an impact on vaccine efficacy and disease outcome. The reason for selecting C57BL/6 for this study is based on our interest in elucidating the role of Toll-like receptors (TLR) in the innate immune response against *Francisella* and vaccine candidates against tularemia, as most TLR knock-out mouse strains are available in this genetic background. The high level of protection observed was associated with progressive weight recovery and a pronounced decrease in bacteremia (10 to 100-fold) in the LPS+rPorB group. This result contrasted with control groups, in particular mice that were mock-vaccinated with PBS that exhibited considerable disseminated disease. Interestingly, mice vaccinated with LPS alone also showed decreased levels of bacteremia. One plausible explanation for this phenomenon is that other mechanisms other than bacterial clearance, including lung inflammation or injury, might be contributing to the difference in survival but not in bacterial loads between LPS- and LPS+rPorB-vaccinated mice. Serum from mice vaccinated with LPS+rPorB showed an overall increased production of antigen-specific antibodies, compared with antibody levels in sera from the LPS-, rPorB- and PBS-immunized groups, which presented low levels of class switched, LPS-specific immunogloblulins. For both antigen specific IgG and IgM we observed that some mice were more responsive than others, indicating variability in the humoral response to LPS administered in combination with rPorB. Nasal vaccination is known to induce serum IgG due to the capacity of mucosal immune cells (e.g. DCs, B cells) to activate and effectively mediate contact with systemic immune sites [35]. In our model, mice presented variable levels of antigen specific antibodies and the higher amounts did not necessarily correlate with protection from challenge. One plausible explanation is that despite the low levels detected, these antibodies may still have a higher affinity for our antigen, and this may potentially be associated with the protective immune response observed. Relative affinity assays will be conducted in future studies. Another possibility is that antibodies are produced and rapidly consumed locally, explaining the containment of the infection in the lung. Other factors, like cellular immunity, might also be playing a crucial role in conferring protection against respiratory challenge. We were able to detect dendritic cells (DCs), follicular dendritic cells (FDCs) and macrophages within the lymphoid structures of the murine lungs, suggesting a role for this cell type in cell mediated immunity in response to our intranasal vaccine and challenge regimen. Recently, another study reported a role for DCs in iBALT homeostasis and induction of antibody responses [36].

In this study we report the development of organized lymphoid structures forming in peribronchial and perivascular areas of lungs following intranasal vaccination and challenge in a murine model of experimental tularemia. In a previous report, organized lymphoid areas defined as inducible BALT (iBALT) developed in the lungs of C57BL/6 mice following infection with the Influenza virus [27]. Lymphoid aggregates were also observed in various other animal species after infection with *Mycobacterium tuberculosis*, *Pseudomonas aeruginosa* and *Mycoplasma* spp. [30]–[32]. In human lungs, organized lymphoid areas have been described in patients with pulmonary complications of autoimmune diseases including rheumatoid arthritis and Sjogren syndrome [34], or other chronic lung conditions like hypersensitivity bronchiolitis and pan-bronchiolitis [37], [38]. In *Francisella tularensis* infection studies, one group found that LVS administered via the aerosol route induced medium sized lymphoid aggregates in murine lungs [39]. The authors hypothesized that these clusters might either be residual inflammatory foci, or *Francisella*-specific T cells to be recalled upon re-infection [39]. Similarly, our group described the appearance of peribronchial lymphoid aggregates in mice that spontaneously recovered from experimentally induced tularemia 50 days after intranasal infection with 10^4^ CFU (10× LD_50_) of LVS [40]. In the present model, we observed that lungs of mice vaccinated intranasally with LPS+rPorB candidate presented accumulations of lymphoid cells around the bronchi and vessels of the lung parenchyma. For simplicity we will refer to these structures as BALT, although they are clearly distinct from the follicles constitutively present in the lungs of certain animal species. Immunofluorescence analysis of the follicles revealed highly organized structures in the lungs of the mice immunized with the LPS+rPorB mice and challenged with LVS. The presence of dense B cell areas containing proliferating B cells and germinal center B cells, surrounded by less dense T cell infiltrates, in vaccinated/challenged survivors, were indicative of BALT formation. In contrast, scattered and disorganized B and T cell clusters were detected in lung tissue of control groups. Additional immunological factors induced by nasal vaccination and bacterial challenge were analyzed in BALT areas. Lung sections were stained for local antibody production and we found that IgG, IgA and IgM producing plasma cells were contained within BALT structures. IgG antibody producing plasma cells were more abundant than IgM+ and IgA+ cells. Given that some of them were positive for the cell proliferation marker PCNA, this may indicate that they are being directly stimulated by antigen. Considering that DCs were also present in BALT structures, it is also possible that these immune cells can induce factors like B cell-activating factor (BAFF), a proliferating inducing ligand (APRIL) and interleukin-6 (IL-6) that may promote expansion and survival of plasma cells [41], [42]. According to the location of the PCNA clusters, it is likely that plasma cells are being generated in germinal centers (GCs) of BALT areas, and may play an important role in preventing dissemination of infection and promoting accelerated local bacterial clearance. The observation that GC-like structures were not present in draining lymph nodes (data not shown) may suggest that immunity is initiated and maintained more efficiently in the lung. We also observed the local production of homeostatic chemokines that play a pivotal role in the architectural maintenance of tertiary lymphoid structures (CXCL13 and CCL21) inside BALT structures. CXCL13 is thought to be mainly involved in the recruitment of CXCR+ B cells and critical for maintaining B cell follicle organization. In contrast, CCL21 attracts CCR7+ T cells and dendritic cells at the infection site. Attraction of naïve CCR7+ cells, CCR7+ dendritic cells and central memory T cells could contribute to accelerated T cell priming and fast activation of memory T cells, enhancing the cellular immune response in the lung of vaccinated mice, and promoting faster bacterial clearance. CCL21 was also detected by ELISA in lung lysates and serum from mice euthanized at day 30 post-challenge. Increased chemokine levels were detected in the LPS+rPorB vaccinated/challenged group, similarly to previously reported studies on chronic tuberculosis infection and autoimmunity [30], [43]. Overall our findings may suggest that local antibody production and cell mediated immunity, activated by vaccination with LPS+rPorB and further stimulated by LVS challenge, triggered the formation of BALT in an attempt to clear bacterial infection in the lung. As mentioned above, infection with LVS alone also induced accumulation of lymphocytes around bronchi [40], but after IF staining we observed a lower cellular organization of the areas (data not shown). This indicates that repetitive intranasal stimulation (vaccination) followed by challenge with LVS contributed to an increased organization of the follicles facilitating local activation of cells from the innate (macrophages, dendritic cells) and adaptive immune system (B cells and T cells). Our results support a potential beneficial role of BALT in the host infected with *F. tularensis*. In humans the role of BALT is unknown, and it is still unclear whether newly induced organized lymphoid follicles are either beneficial or detrimental for the host [44]. Most adult healthy human lungs present little evidence of these structures [24], but similar areas have been previously found in patients with pulmonary conditions, infectious diseases like tuberculosis and complications of autoimmune disease, where iBALT plays a role in lung immunopathology [34], [37], [45]. Since the structures we find in the current study appear in mice surviving infection when bacteria are cleared from the target organs, the direct translational significance of our findings into human immunity is presently difficult to define. Interesting future studies that could support our findings in the mouse model may include analysis of BALT in lung tissue from patients who have recovered versus those who have died from respiratory tularemia. Overall, it is important to keep in mind the diversity of lung lymphoid structures (classic BALT versus iBALT) in different physiological and pathological settings, including autoimmunity, long lasting antigenic stimulation and chronic bacterial disease [46], as well as the different animal species they are detected in, for a better understanding of their role in the induction of local protective immunity.

One additional interesting finding was the high stability of BALT that was detected at late time points after intranasal challenge in survivor mice, contrasting with the absence of lymphoid aggregates in lungs from moribund mice (data not shown). Although we showed pictures of BALT taken at 30 days post-infection, these organized structures were still found at 120 days after infection. Bacterial cultures of lung lysates were negative at these late time points, suggesting that factors other than chronic infection must play a role in BALT persistence. Similarly, another study reported that iBALT persisted in murine lungs even after the Influenza virus had apparently been cleared [47]. One explanation for this phenomenon is that protein and peptide antigens could remain in these areas for extended periods, continuously stimulating B and T cells, sustaining local antibodies for prolonged periods of time [48], [49]. Spleens and livers from immunized and subsequently challenged mice also did not contain any viable *F. tularensis*. This may suggest that immune responses initiated in the lungs are also able to control disseminated infection, leading to bacterial clearance from peripheral tissues. It should be noted that the studies we mentioned above addressed potential BALT persistence in viral infection, while in the case of bacteria lymphoid structures have mainly been associated with chronic disease, as in the case of *M. tuberculosis* and *P. aeruginosa* [30], [32]. In our *F. tularensis* vaccine/challenge model it is difficult to exactly conclude what contributes to BALT maintenance over time, though its development in lungs of mice surviving tularemia suggests that it may play a role in the induction of local immunity and protection in our model of experimental tularemia.

In the present work, we demonstrate the efficacy of *F. tularensis* LPS, combined with meningococcal PorB against experimental tularemia. For future studies it would be optimal to test the adjuvanticity of this protein when combined with other promising antigen candidates, and test their efficacy against LVS and virulent strains of *F. tularensis*. We also report the association of lung BALT structures with mouse survival following vaccination and subsequent respiratory infection. Additional studies are required to reveal the mechanisms involved in BALT formation and its active role in the induction of local and systemic protection against pulmonary *Francisella* infection.

## Materials and Methods

### Bacterial strains and growth conditions

*F. tularensis* subsp. *holarctica* live vaccine strain (LVS- ATCC 29684) was obtained from the CDC, Fort Collins, CO. For mouse challenge, LVS was grown on chocolate agar for 72 hours and bacterial suspensions were prepared by resuspending colonies in PBS at an OD_600_ of 0.3 [12], [40].

### Purification methods

Lipopolysaccharide (LPS) was isolated from the LVS strain using the hot phenol method as previously described [12]. Purification of recombinant PorB (rPorB) was performed based on a well established protocol [50]. *E. coli* BL21(λDE3) *ΔompA*, obtained from Dr. Milan Blake at the Rockefeller University, was grown on a LB agar plate containing 50 µg/ml kanamycin and incubated overnight at 37°C. Colonies from the plate were inoculated into 10 ml of liquid medium consisting of supplemented M9 minimal media grown overnight at 37°C. Subsequent growth was induced with IPTG for 3 hours at 37°C. After centrifugation the bacterial pellet was resuspended in 3 ml TEN buffer. PMSF and lysozyme were added, followed by deoxycholate and the suspension was then placed in a 37°C water bath. DNase I was then added to the mixture. The lysate was centrifuged at 10,000×*g* for 20 min at 4°C. The pellet was resuspended in 5 ml TEN buffer and then sonicated. Zwittergent (10%, wt/vol) was added to the mixture, sonicated for 10 min and loaded onto Sephacryl S-300 gel filtration columns previously equilibrated with 100 mM Tris, 200 mM NaCl, 10 mM EDTA, pH 8.0. The flow rate of the column was set at 0.8 ml/min and 10 ml fractions were collected. Fractions containing protein as determined by measurement of OD_280_ were analyzed by SDS-PAGE.

### Preparation of proteosomes and SDS-PAGE

Recombinant PorB proteosomes were prepared as previously described [51]. Briefly, fractions containing porin were pooled and precipitated with 80% (v/v) ethanol and held at −20°C overnight. The precipitate was centrifuged at 15,000×*g* for 20 min, the pellet was resuspended in 10 ml 10 mM Hepes buffer containing 10% D-octyl-glucoside (DOG), pH 7.2 and dialyzed extensively against PBS containing 0.02% sodium azide [51]. The protein concentration was determined using a BCA protein assay kit (Pierce) and tested for endotoxin by Pyrotell (Associates of Cape Cod, East Falmouth, MA) with a sensitivity of 0.06 endotoxin units per milliliter (EU/ml). Fractions containing protein as determined by measurement of OD_280_ were analyzed by SDS-PAGE. Precise™ Protein Gels 4%–20% were used in Tris-HEPES-SDS buffer (Pierce Biotechnology, Inc.) and were stained with standard Coomassie (Bio-Rad, Inc. Hercules, CA).

### Mice

Seven week old female C57BL/6 mice were obtained from Jackson Laboratories (Bar Harbor, ME) and maintained within the Laboratory Animal Science Center (LASC) at Boston University. All experimental procedures were in accordance with institutional animal care and use committee.

### Intranasal vaccination and challenge

Groups of five to ten mice were immunized by the intranasal (i.n.) route three times at two-week intervals. Mice were anesthetized via the i.p. route with ketamine HCl (Fort Dodge Animal Health, Fort Dodge, IA) and xylazine (Lloyd Laboratories, Shenandoah, IA), and vaccinated with LVS-LPS, LVS-LPS with neisserial rPorB, rPorB alone, or PBS in a total volume of 20 µl (a quantity of 10 µg for each substance). Three to ten mice per group, were bled by the submandibular vein after the last vaccination, to detect the production of LPS-specific immunoglobulins. Four weeks after the last immunization, mice were challenged intranasally with either 10^5^ or 10^6^ CFU of LVS as described before [12]. All animals were closely observed until completely awake from anesthesia. Survival and changes in body weight were recorded for up to 21 days after infection. For lung tissue analysis, survivors were humanely euthanized 30 days post-challenge. Data reported in this study are representative of three independent experiments.

### Antibody and chemokine assays

Mouse serum and lungs were processed for the detection of immune markers. Analysis of LPS-specific serum IgG, IgM and IgA was performed in pre-challenge serum by enzyme-linked immunosorbent assay (ELISA) as previously reported [12], [40]. Briefly, plate wells were coated with LPS from *F. tularensis* LVS (0.25 µg/ml), incubated at 37°C and stored overnight at 4°C. Sera were diluted starting from 1∶50, added to the previously coated wells, and incubated at 37°C. Alkaline phosphatase-conjugated anti-mouse IgG, IgM or IgA (Sigma, St. Louis, MO) were added. After washing, the color was developed with one-step *p*-nitrophenyl phosphate (Pierce, Rockford, IL) and the optical density at 405 nm was measured on an ELx800 reader (Bio-Tek Instruments, Inc., Winooski, VT). Colorimetric values were converted to nanograms/milliliter, according to the standard curves generated for IgG, IgM and IgA. Levels of the CCL21 chemokine were measured in both serum and lung lysates of vaccinated/challenged mice by ELISA using the DuoSet Development System kit specific for CCL21/6Ckine (R&D Systems, Minneapolis, MN).

### Histopathological and morphometric analyses of lymphocytic infiltration

All histopathological procedures were conducted as previously described [40]. Briefly, lungs were removed aseptically 30 days post challenge, and inflated with 10% buffered formalin through the trachea. Tissue was fixed in formalin, embedded in paraffin, and sections were stained with standard hematoxylin and eosin (H&E). Lymphocytic infiltration was analyzed by researchers ‘blinded’ to sample identity, and morphometry was determined using a tool of an Axioplan microscope (Zeiss). The lymphoid areas were defined by the squared micron value for each cluster of lymphocytes encountered in the lung parenchyma. Total area covered by lymphoid follicles was calculated by adding the individual areas occupied by all the lymphoid follicles present in the whole lung section and expressed in squared microns (µm^2^).

### Immunofluorescence and morphometric analysis of BALT

Formation of germinal centers, B cell interaction with T cells, and B cell proliferation were evaluated with a combination of commercial antibodies. B cells were detected with biotinylated antibodies against B220 (clone: RA3-6B2; BD Pharmingen, San Diego, CA) followed by the addition of Streptavidin-Alexa Fluor 488 (Molecular probes, Eugene, OR) or Streptavidin-Alexa fluor 594 (Molecular probes, Eugene, OR). For detecting T cells (CD3-epsilon, clone: M-20; Santa Cruz Biotechnology, Santa Cruz, CA) and proliferating cell nuclear antigen (PCNA, clone C-20; Santa Cruz Biotechnology, Santa Cruz, CA) slides were first incubated with purified goat-anti mouse CD3 and goat anti PCNA. In a second step, CD3 and PCNA were visualized with donkey anti-goat, conjugated to Alexa Fluor 594 (Molecular probes, Eugene, OR). Germinal center B cells were detected by incubating slides with peanut agglutinin-FITC (PNA-FITC) (SIGMA, St. Louis, MO), followed by rabbit anti-FITC, conjugated to Alexa Fluor 488 (Molecular probes, Eugene, OR). Dendritic cells were detected with a combination of hamster-anti mouse CD11c-PE (clone HL3, BD Pharmingen, San Diego, CA) and rat-biotin anti mouse CD11b (M1/70, BD Pharmingen, San Diego, CA), followed by incubation with rabbit anti-PE (Rockland Immunochemicals for Research Inc, Gilbertsville, PA) and Streptavidin FITC (BD Pharmingen, San Diego, CA) and finally PE was detected with anti-rabbit Cy-3 (Jackson Immunoresearch, West Grove, PA). For detecting immunoglobulin producing cells, lung sections were stained with a combination of antibodies directed against goat anti PCNA (Santa Cruz Biotechnology, clone C-20), biotin, rat anti IgM (BD Pharmingen), rat anti-IgA (BD Pharmingen, clone C-10) or FITC-donkey anti-mouse IgG (Jackson Immunoresearch). For T cells we used goat anti CD3 (Santa Cruz Biotechnology, clone M20), and we combined it with anti-PCNA antibody for the identification of proliferating T cells. Anti-PCNA specifically stains nuclei (red), while anti-CD3 stains the cell surface (red), allowing the distinction between the two signals. Goat antibodies were detected with donkey anti-goat, Alexa fluor 568 (Invitrogen) and rat antibodies were detected with donkey anti-rat, Alexa fluor 488 and streptavidin-alexa fluor 488 (Invitrogen). FITC signal was amplified with rabbit anti-FITC, alexa fluor 488. Homeostatic chemokines were detected with goat anti mouse CXCL13 and goat anti mouse CCL21. Follicular dendritic cell (FDC) networks were detected with antibodies against FDCM-1 (BD Pharmingen) and biotin- rat anti mouse CD21-CD35 antibodies. B cells were stained with APC-rat anti CD45R (BD Pharmingen). To visualize chemokines and cells, slides were incubated with donkey anti-goat, conjugated to alexa fluor 568 and with donkey anti-rat, alexa fluor 488 and streptavidin-alexa fluor 488 (Invitrogen). Slow fade gold antifade with DAPI (Molecular probes, Eugene, OR) was used to counterstain tissues and to detect nuclei. Images were obtained with a Zeiss Axioplan 2 microscope and recorded with a Zeiss AxioCam digital camera. Whole lungs from four mice per group underwent morphometric analysis in a blinded manner using the morphometric tool of Zeiss Axioplan microscope (Zeiss), which determines the area defined by the squared pixel value for each B cell follicle. Total area occupied by B cell follicles was calculated as mentioned above.

### Statistical analysis

Differences between experimental groups were determined by the Mann-Whitney U nonparametric test by using GraphPad Prism software version 4.02 (San Diego, CA). *P* values ≤0.05 were considered significant, while *P* values ≤0.01 were considered highly significant.

## References

[1] Di Tommaso A, Saletti G, Pizza MG, Rappuoli R, Dougan G (1996). Induction of antigen-specific antibodies in vaginal secretions by using a nontoxic mutant of heat-labile enterotoxin as a mucosal adjuvant.. Infect Immun.

[2] Hirabayashi Y, Kurata H, Funato H, Nagamine T, Aizawa C (1990). Comparison of intranasal inoculation of influenza HA vaccine combined with cholera toxin B subunit with oral or parenteral vaccination.. Vaccine.

[3] Ciabattini A, Giomarelli B, Parigi R, Chiavolini D, Pettini E (2008). Intranasal immunization of mice with recombinant *Streptococcus gordonii* expressing NadA of *Neisseria meningitidis* induces systemic bactericidal antibodies and local IgA.. Vaccine.

[4] Wu HY, Russel MW (1993). Induction of mucosal immunity by intranasal application of a streptococcal surface protein antigen with the cholera toxin subunit B.. Infect Immun.

[5] Neutra RM, Kozlowski PA (2006). Mucosal vaccines: the promise and the challenge.. Nat Rev Immunol.

[6] Chen H (2000). Recent advances in mucosal vaccine development.. J Control Release.

[7] Wu TH, Hutt JA, Garrison KA, Berliba LS, Jhou Y (2005). Intranasal vaccination induces protective immunity against infection with virulent *Francisella tularensis* biovar A.. Infect Immun.

[8] Pammit MA, Raulie EK, Lauriano CM, Klose KE, Arulanandam BP (2006). Intranasal vaccination with a defined attenuated *Francisella novicida* strain induces gamma interferon-dependent antibody-mediated protection against tularemia.. Infect Immun.

[9] Bakshi CS, Malik M, Mahawar M, Kirimanjeswara G, Hazlett KRO (2008). An improved vaccine for prevention of respiratory tularemia caused by *Francisella tularensis* SchuS4 strain.. Vaccine.

[10] Pechous RD, McCarthy TR, Mohapatra NP, Soni S, Penoske RM (2008). A *Francisella tularensis* Schu S4 Purine auxotroph is highly attenuated in mice but offers limited protection against homologous intranasal challenge.. PLoS ONE.

[11] Oyston PCF, Quarry JE (2005). Tularemia vaccine: past, present and future.. Antonie Leeuwenhoek.

[12] Chiavolini D, Weir S, Murphy JR, Wetzler LM (2008). *Neisseria meningitidis* PorB, a Toll-Like Receptor 2 Ligand, Improves the Capacity of *Francisella tularensis* Lipopolysaccharide To Protect Mice against Experimental Tularemia.. Clin Vaccine Immunol.

[13] Conlan JW, Shen H, Webb A, Perry MB (2002). Mice vaccinated with the O-antigen of *Francisella tularensis* LVS lipopolysaccharide conjugated to bovine serum albumin develop varying degrees of protective immunity against systemic or aerosol challenge with virulent type A and type B strains of the pathogen.. Vaccine.

[14] Conlan JW, Vinogradov E, Monteiro MA, Perry MB (2003). Mice intradermally-inoculated with the intact lipopolysaccharide, but not the lipid A or O-chain, from *Francisella tularensis* LVS rapidly acquire varying degrees of enhanced resistance against systemic or aerogenic challenge with virulent strains of the pathogen.. Microb Pathog.

[15] Dreisbach VC, Cowley SC, Elkins KL (2000). Purified lipopolysaccharide from *Francisella tularensis* live vaccine strain (LVS) induces protective immunity against LVS infection that requires B cells and gamma interferon.. Infect Immun.

[16] Fulop M, Mastroeni P, Green M, Titball RW (2001). Role of antibody to lipopolysaccharide in protection against low- and high-virulence strains of *Francisella tularensis*.. Vaccine.

[17] Cole LE, Yang Y, Elkins KL, Fernandez ET, Qureshi N (2009). Antigen-specific B-1a antibodies induced by *Francisella tularensis* LPS provides long-term protection against *F. tularensis* LVS challenge.. PNAS.

[18] Fulop M, Manchee R, Titball RW (1995). Role of lipopolysaccharide and a major outer membrane protein from *Francisella tularensis* in the induction of immunity against tularemia.. Vaccine.

[19] Golovliov I, Ericsson M, Akerblom L, Sandstrom G, Tarnvik A (1995). Adjuvanticity of ISCOMs incorporating a T cell-reactive lipoprotein of the facultative intracellular pathogen *Francisella tularensis*.. Vaccine.

[20] Hartley MG, Green M, Choules G, Rogers D, Rees DG (2004). Protection afforded by by heat shock protein 60 from *Francisella tularensis* is due to copurified lipopolysaccharide.. Infect Immun.

[21] Huntley JF, Conley PG, Rasko DA, Hagman KE, Apicella MA (2008). Native outer membrane proteins protect mice against pulmonary challenge with virulent type A *Francisella tularensis*.. Infect Immun.

[22] Gregory SH, Chen WH, Mott S, Palardy JE, Parejo NA (2010). Detoxified Endotoxin Vaccine (J5dLPS/OMP) Protects Mice Against Lethal Respiratory Challenge with *Francisella tularensis* SchuS4..

[23] Bitsaktsis C, Rawool DB, Li Y, Kurkure NV, Iglesias B (2009). Differential requirements for protection against mucosal challenge with *Francisella tularensis* in the presence versus absence of cholera toxin B and inactivated *F. tularensis*.. J Immunol.

[24] Tschernig T, Pabst R (2000). Bronchus-asscoated lymphoid tissue (BALT) is not present in the normal adult lung but in different diseases.. Pathobiology.

[25] Bienenstock J, Johnston N, Perey DY (1973). Bronchial lymphoid tissue. I. Morphologic characteristics.. Lab Invest.

[26] Bienenstock J, Johnston N, Perey DY (1973). Bronchial lymphoid tissue. II. Functional characteristics.. Lab Invest.

[27] Moyron-Quiroz JE, Rangel-Moreno J, Kusser K, Hartson L, Sprague F (2004). Role of inducible bronchus associated lymphoid tissue (iBALT) in respiratory immunity.. Nat Med.

[28] Bienenstock J, McDermott MR (2005). Bronchus- and nasal-associated lymphoid tissues.. Immunol Rev.

[29] Rangel-Moreno J, Moyron-Quiroz JE, Carragher D, Randall TD (2007). Role of lymphotoxin and homeostatic chemokines in the development and function of locale lymhpid tissue in the respiratory tract.. Immunologia.

[30] Kahnert A, Hopken UE, Stein M, Bandermann S, Lipp M (2007). *Mycobacterium tuberculosis* triggers formation of lymphoid structure in murine lungs.. J Infect Dis.

[31] Rodriguez F, Fernandez A, Oros J, Ramirez AS, Luque R (2001). Changes in lymphocyte subsets in the bronchus-associated lymphoid tissue of goats naturally infected with different *Mycoplasma* species.. J Vet Med B Infect Dis Vet Public Health.

[32] Iwata M, Sato A (1991). Morphological and Immunohistochemical Studies in the Lungs of and Bronchus-Associated Lymphoid Tissue in a Rat Model of Chronic Pulmonary Infection with *Pseudomonas aeruginosa*.. Infect Imm.

[33] Chen HD, Fraire AE, Joris I, Welsh RM, Selin LK (2003). Specific history of heterologus virus infections determines anti-viral immunity and immunopathology in the lung.. Am J Pathol.

[34] Rangel-Moreno J, Hartson L, Navarro C, Gaxiola M, Selman M (2006). Inducible bronchus-associated lymphoid tissue (iBALT) in patients with pulmonary complications of rheumatoid arthritis.. J Clin Invest.

[35] Csaba N, Garcia-Fuentes M, Alonso MJ (2009). Nanoparticles for nasal vaccination.. Adv Drug Deliv Rev.

[36] GeurtvanKessel CH, Willart MA, Bergen IM, van Rijt LS, Muskens F (2009). Dendritic cells are crucial for maintenance of tertiary lymphoid structures in the lung of influenza virus-infected mice.. J Exp Med.

[37] Suda T, Chida K, Hayakawa H, Imokawa S, Iwata M (1999). Development of Bronchus-Associated Lymphoid Tissue in Chronic Hypersensitivity Pneumonitis.. Chest.

[38] Sato A, Chida K, Iwata M, Hayakawa H (1992). Study of bronchus associated lymphoid tissue in chronic in patients with diffuse panbronchiolitis.. Am Rev Respir Dis.

[39] Conlan JW, Shen H, KuoLee R, Zhao X, Chen W (2005). Aerosol-, but not intradermal-immunization with the live vaccine strain of *Francisella tularensis* protects mice against subsequent aerosol challenge with a highly virulent type A strain of the pathogen by an alphabeta T cell- and interferon gamma- dependent mechanism.. Vaccine.

[40] Chiavolini D, Alroy J, King CA, Jorth P, Weir S (2008). Identification of immunologic and pathologic parameters of death versus survival in respiratory tularemia.. Infect Immun.

[41] Mohr E, Serre K, Manz RA, Cunningham AF, Khan M (2009). Dendritic cells and monocyte/macrophages that create the IL-6/APRIL-Rich lymph node microenvironments where plasmablasts mature.. J Immunol.

[42] Randall TD (2010). Pulmonary dendritic cells: thinking globally, acting locally.. J Exp Med.

[43] Rangel-Moreno J, Moyron-Quiroz JE, Hartson L, Kusser K, Randall TD (2007). Pulmonary expression of CXC chemokine ligand 13, CC chemokine ligand 19, and CC ligand 21 is essential for local immunity to influenza.. PNAS.

[44] Tschernig T, Pabst R (2009). What is the clinical relevance of different lung compartments?. BMC Pulm Med.

[45] Ulrichs T, Kosmiadi GA, Trusov V, Jorg S, Pradl L (2004). Human tuberculous granulomas induce peripheral lymphoid follicle-like structures to orchestrate local host defence in the lung.. J Pathol.

[46] Pabst R (2007). Plasticity and heterogeneity of lymphoid organs. What are the criteria to call a lymphoid organ primary, secondary or tertiary?. Immunol Lett.

[47] Moyron-Quiroz JE, Rangel-Moreno J, Hartson L, Kusser K, Tighe MP (2006). Persistence and responsiveness of immunologic memory in the absence of secondary lymphoid organs.. Immunity.

[48] Turner DL, Cauley LS, Khanna KM, Lefrancois L (2007). Persistent antigen presentation after acute vesicular stomatitis virus infection.. J Virol.

[49] Jelley-Gibbs DM, Brown DM, Dibble JP, Haynes L, Eaton SM (2005). Unexpected prolonged presentation of influenza antigens promotes CD4 T cell memory generation.. J Exp Med.

[50] Qi HL, Tai JY, Blake MS (1994). Expression of large amounts of neisserial porin proteins in *Escherichia coli* and refolding of the proteins into native trimers.. Infect Immun.

[51] Massari P, King CA, MacLeod H, Wetzler LM (2005). Improved purification of native meningococcal porin PorB and studies on its structure/function.. Protein Expr Purif.

